# Thermotherapy Treatments of Fruit Trees of the Genus Prunus Infected by Viruses and Bacteria

**DOI:** 10.3390/plants15162440

**Published:** 2026-08-11

**Authors:** Natàlia Puig-Gay, Carlos Rieder, Mireia Bordas

**Affiliations:** Agromillora Iberia SLU, El Rebato s/n, Subirats, 08739 Barcelona, Spain; npuig@agromillora.com (N.P.-G.); crieder@agromillora.com (C.R.)

**Keywords:** *Prunus* sp., thermotherapy, *Xanthomonas arboricola pv. pruni*, *Prunus necrotic ringspot virus*, hot water treatment

## Abstract

The thermotherapy technique has been used to evaluate the viability of hot water treatments (HWTs) for the elimination of different pathogens that infect commercial fruit trees. The present project focuses on common pathogens of *Prunus* species at the nursery level when external propagation material is needed, which is not always in optimal sanitary conditions. Different combinations of temperature/duration treatments were applied to budwood of nectarines infected by *Xanthomonas arboricola pv. pruni* (*Xap*) and almonds infected by *Prunus necrotic ringspot virus* (*PNRSV*). These treated buds were then grafted onto disease-free micropropagated rootstocks. In bacteria-infected nectarine tissues treated at 45 °C for 40 min, *Xap* was not detected in any sample, preserving a high viability of the grafted plants. In virus-infected almond tissues treated at 45 °C for 50 min a few individual plants were identified in which no virus was detected. These results demonstrated the effectiveness of the HWT and suggest that these techniques could be applied at nursery level, on a large scale for the eradication of bacteria or on a small scale for the recovery of virus-free individuals.

## 1. Introduction

Plant pathogens are causing significant economic losses and impacting biodiversity loss across all ecosystems. The damage caused by the quarantine plant pathogenic bacterium *Xylella fastidiosa* (*Xf*) in Europe is an example of the devastating consequences and impact on agriculture. The study described here has been carried out within the framework of the Bexyl (Beyond Xylella project) with the aim of mitigating the impact of Xylella in Europe (HORIZON-CL6-2021-FARM2FORK-01-04, grant number 101060593). Although the project focuses on Xylella, these protocols have also been adapted for use with other important bacterial and viral pathogens, most of which are introduced into production systems through the incorporation of external plant material.

In nurseries for the propagation of fruit trees, it is an essential requirement that the planting material meets high sanitary standards. With regard to vegetative propagation, mother plants used to produce propagation material are critical, and it is advisable to have ‘propagation stocks’ maintained under conditions ensuring freedom from infection. The same would apply to clonal propagation laboratories, which initiate in vitro cultures from pathogen-free plants.

In the nursery stage, the problem arises when it is necessary to use external propagation material, which is not always in optimal sanitary conditions. This would happen in the case of grafted plants with scions sent by the customer sometimes directly from the breeder. If we set these high standards of sanitary quality, after checking and verifying infections, these plant materials would be discarded, which represents a great loss for the producer.

Attempts to sanitize pathogen-infected plants have yielded positive results, although they are not always easy to apply on a large scale in nursery farms [1,2].

Dziedzic [3] succeeded in eliminating *Prunus necrotic ring spot virus* (*PNRSV*) from plum ‘Earliblue’ through thermotherapy in vitro using several temperature regimes (36–38 °C) and treatment durations (10 to 29 days). This technique proved effective for virus elimination, although it is time-consuming and not applicable to nursery-grown grafted varieties.

A relatively easy method for eradicating pathogens from propagation material is hot water treatments in vivo. In grapes, dormant buds are submersed in a hot water bath at 50 °C for 30 min and experimental evidence has shown that this method can be effective for *Agrobacterium tumefaciens* Biovar 3 [4].

To assess the potential of this technique in *Prunus* spp., we have plan to conduct large-scale trials with a greater number of varieties and pathogens (bacteria and viruses), to attempt their eradication and clarify the treatment effects on the viability and growth of plants in the nursery, as well as the effect in the field.

The objective of this study was to test the effectiveness of hot water treatment in dormant plant material as a method of *PNRSV* and *Xap* eradication in *Prunus* spp. and to evaluate its applicability as a technique in nurseries integrated into routine operations without entailing excessive additional costs.

## 2. Results

### 2.1. Effect of HWT on the Viability of Prunus spp.

**Figure 1:**
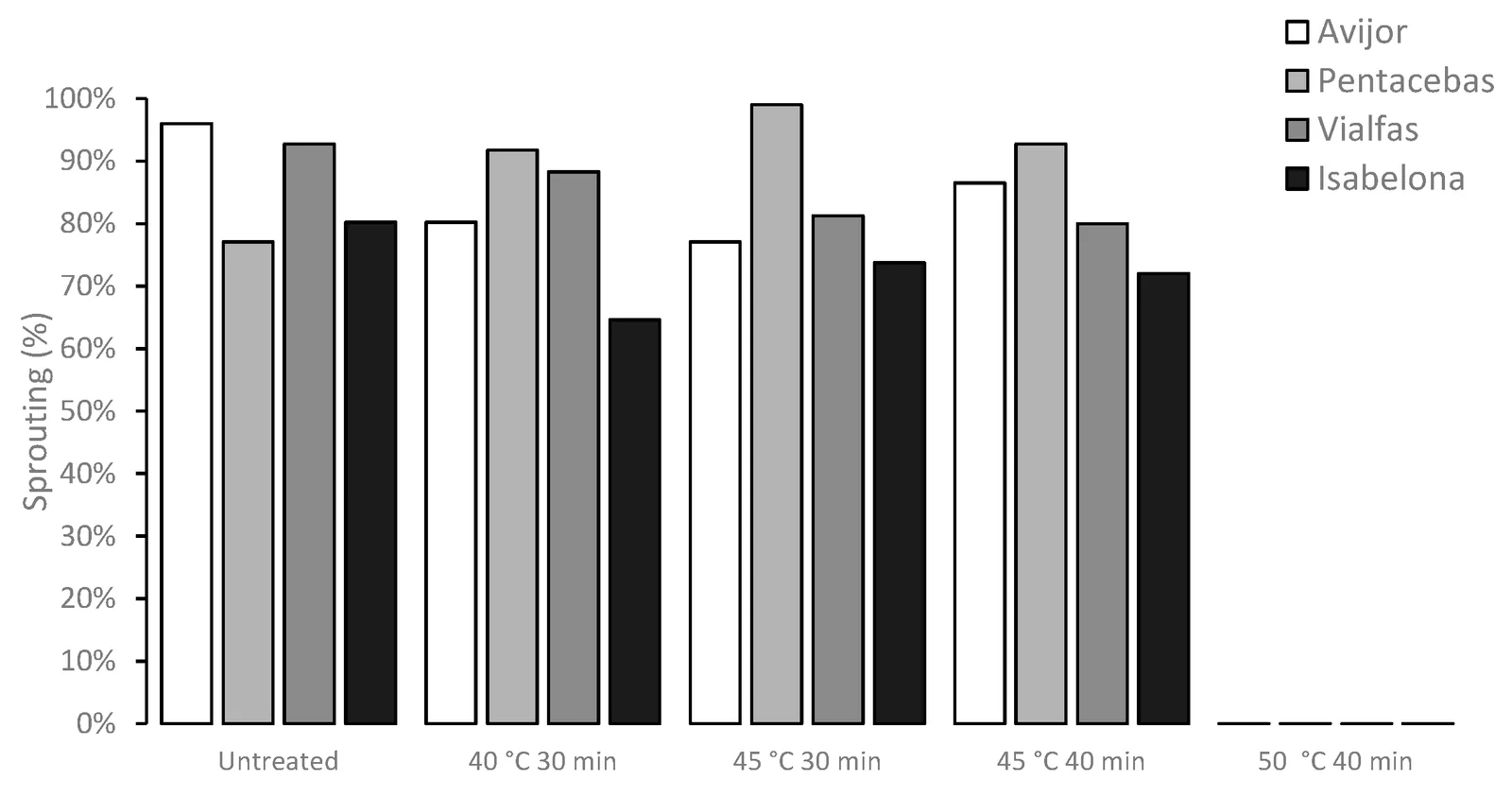
Sprouting percentage (%) of four *Prunus* varieties under different treatments.

In the first test, the viability of untreated branches was over 77% for ‘Pentacebas’, 80% for ‘Isabelona’, over 92% for ‘Vialfas’ and up to 96% for ‘Avijor’, likely depending on the condition of the mother plants. At 45 °C, the impact of the HWT was less significant, and regardless of the soaking time, the success rate was over 70% in all varieties. Furthermore, the observed behavior differed among the four varieties evaluated, with the lowest for ‘Pentacebas’, which reached 99% with a 30 min soaking time. (see Figure 1). None of the buds treated at 50 °C for 40 min sprouted after grafting.

**Figure 2:**
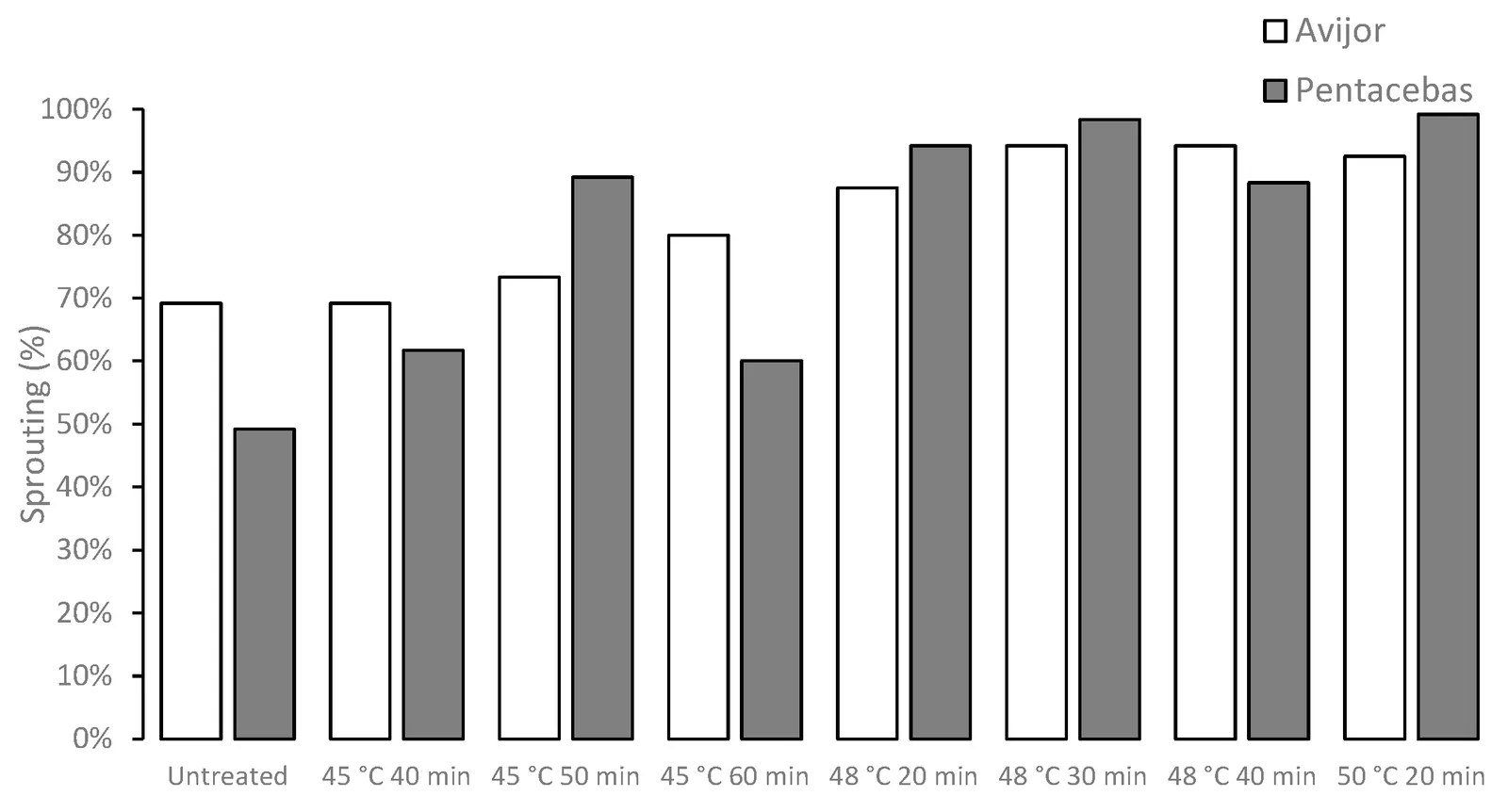
Sprouting percentage in ‘Avijor’ and ‘Pentacebas’ almond under different treatments.

In the second test, the success rate was higher in all buds treated with HWT compared to untreated buds. The highest success rates were observed at 48 °C and 50 °C, with a maximum of 99% for ‘Pentacebas’ with the 50 °C/20 min combination and a maximum of 94% for ‘Avijor’ with 48 °C/40 min combination (see Figure 2). Temperatures close to 48 °C maintain adequate viability levels, improving results compared to lower temperatures.

These trials determined that economically viable sprouting is achieved in the range of 45–50 °C and times between 20 and 40 min, with different levels of tolerance observed depending on the species.

### 2.2. Efficacy of HW Treatments on Prunus spp. and Against Different Pathogens

The previous results showed good survival rates at temperatures between 45 °C/30 min and 50 °C/20 min. We applied two combinations of temperature/duration *Xap*-infected nectarine budwood and three combinations on *PNRSV*- infected almond budwood, both naturally infected plant material collected from commercial mother plant stock.

**Figure 3:**
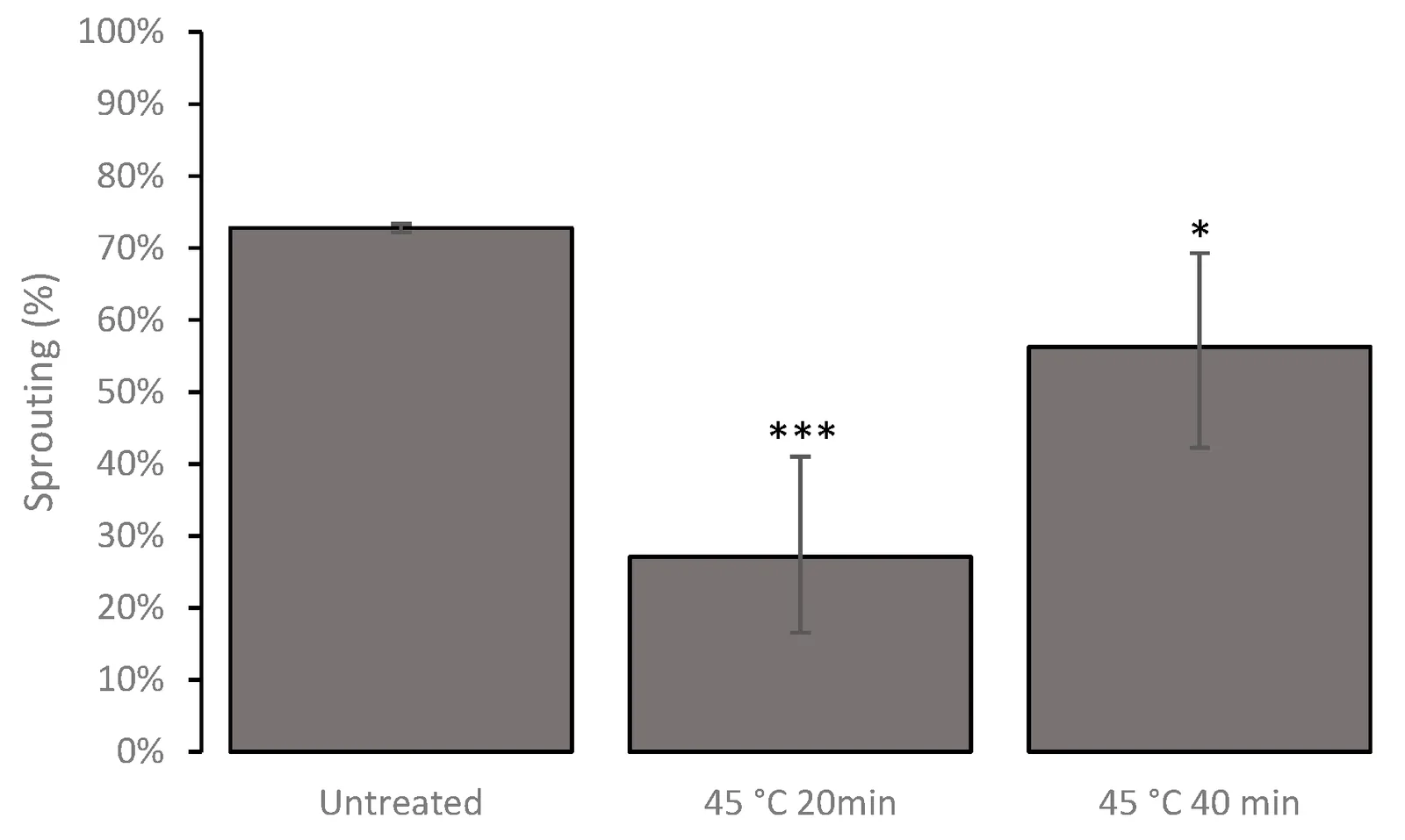
Sprouting percentage (%) by treatment in *Xap*-infected plants. Bars represent observed sprouting percentage (%) with 95% confidence intervals calculated using the Wilson method. Asterisks indicate statistically significant differences compared to the untreated control (Fisher’s exact test with Holm adjustment; * *p* < 0.05; *** *p* < 0.001).

Regarding the nectarines, the sprouting percentage is shown in Figure 3. Untreated branches reached 72% and it is worth noting that the success rate was higher at 45 °C for 40 min (56%) compared to 45 °C for 20 min (27%).

**Figure 4:**
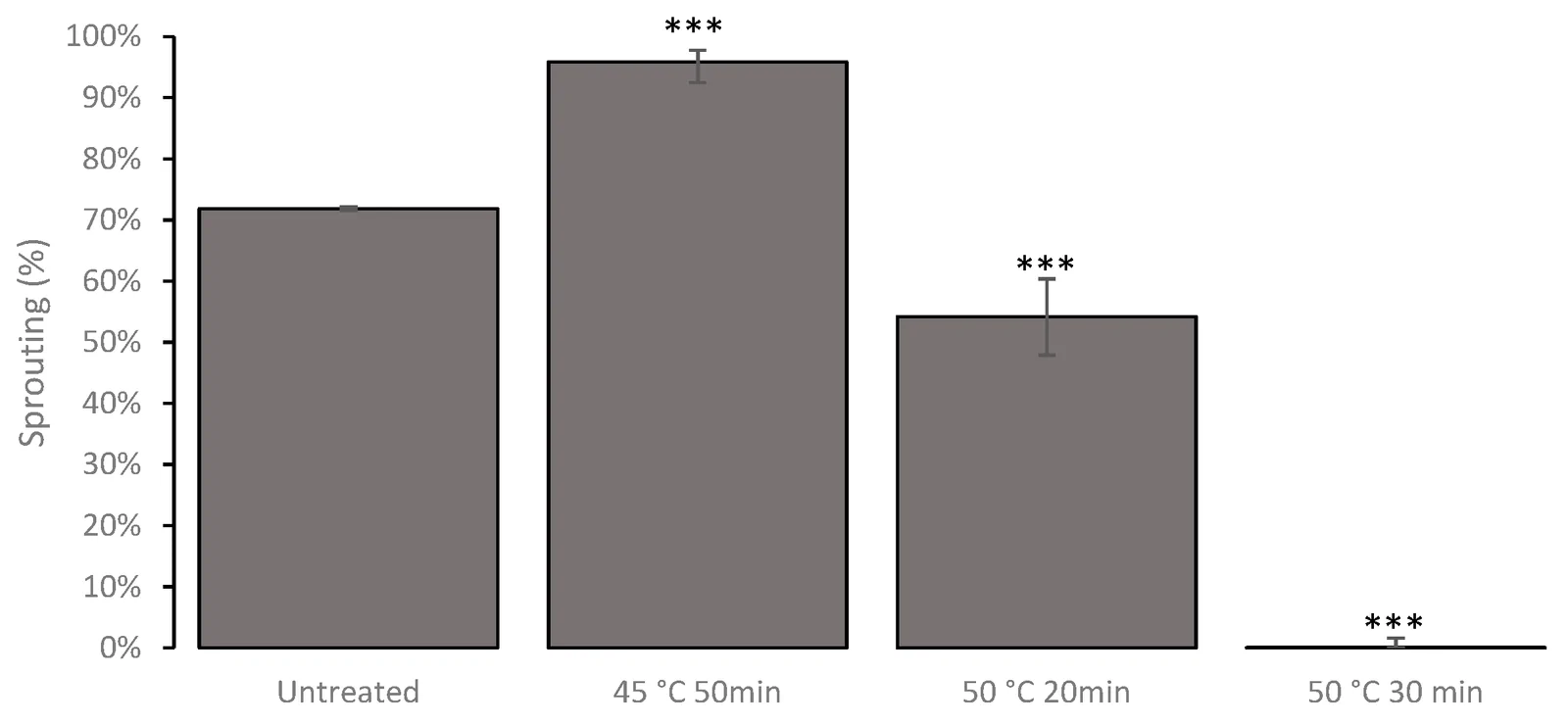
Sprouting percentage (%) by treatment in *PNRSV*-infected plants. Bars represent observed sprouting percentage (%) with 95% confidence intervals calculated using the Wilson method. Asterisks indicate statistically significant differences compared to the untreated control (pairwise comparisons of proportions with Holm adjustment; *** *p* < 0.001).

In almonds, the highest survival rate was obtained using the combination of 45 °C for 50 min, reaching 96%, while no buds sprouted at 50 °C/30 min (see Figure 4).

The effectiveness of the treatment against host–pathogen interactions is shown in Table 1 and Table 2. The samples were collected from all sprouted plants, included in each treatment batch and pooled for analysis.

In infected nectarine buds treated at 45 °C 40 min. *Xap* was not detected. This result demonstrated the effectiveness of this HWT, ensuring both the survival of the grafted material and the elimination of the pathogenic bacteria. Furthermore, the observed survival rates are acceptable for implementation under commercial propagation conditions.

In the infected *PNRSV* almond trial, at 50 °C for 20 min the samples of sprouting plants remained RT-PCR positive and 50 °C for 30 min produced non-sprouting plants, so neither condition achieved useful virus eradication with acceptable plant survival.

However, batches of shoots treated at 45 °C for 50 min tested weakly positive for *PNRSV* by RT-PCR. Consequently, further experiments were conducted using smaller batches. Ten batches were analyzed, and *PNRSV* was not detected by RT-PCR in three of them, whereas the remaining seven yielded Ct values ranging from 23 to 32.

## 3. Discussion

The present study evaluated the effect of hot water treatment (HWT) on the viability of Prunus propagation material under nursery conditions and assessed its potential for reducing infections caused by two economically important pathogens, *Xanthomonas arboricola pv. pruni* (Xap) and *Prunus necrotic ringspot virus* (PNRSV). The results obtained with pathogen-infected material indicate that HWT can be applied while maintaining acceptable levels of bud viability under selected temperature–time combinations and may contribute to pathogen reduction.

The response differed among the *Prunus* materials evaluated, and was influenced by several factors, including varietal effects, the size of the treated plant material, and the conditions and timing of sampling, as also reported by other authors [5,6].

Interestingly, ‘Pentacebas’ exhibited higher sprouting percentages than the other cultivars under several HWT conditions. This response may be related to cultivar-specific differences in bud dormancy status, tissue composition, water content or intrinsec heat tolerance, all of which have previously been suggested to influence thermotherapy outcomes. However, as these factors were not assessed in the present study, the underlying mechanisms remain unclear.

In nectarine material infected with *Xap*, no bacterial DNA was detected through RT-PCR in pooled samples collected from plants treated at 45 °C for 40 min, whereas untreated material tested positive, suggesting that HWT can contribute to pathogen suppression in *Prunus* propagation material under the conditions evaluated.

In almond material infected with *PNRSV*, a limited number of RT-PCR-negative samples were identified following treatment at 45 °C for 50 min. Therefore, this approach cannot be considered suitable for the commercial sanitation of entire batches of plant material in nurseries, although it may have practical value for recovering selected pathogen-free individuals intended for the establishment of mother plants.

Plant thermotherapy has been applied for decades to eliminate viruses in woody plants, most of the work focused on grapevine sanitation, apple, citrus, and other fruit tree species [7,8]. HWT has been described as the most effective treatment against viruses characterized by parenchyma localization, compared with meristem culture technique which is more suitable for phloemic viruses that are limited to vascular tissues [9].

Regarding bacterial pathogens, M. Keck [10] stated that the use of heat (38 °C for 6 days) may be a possible procedure for the treatment of plant propagation material to drastically reduce and in certain circumstances eliminate the population of *Erwinia amylovora* within the plant tissues of pome fruit scions.

The mechanisms by which thermotherapy contributes to pathogen elimination remain incompletely understood. Research aimed at investigating the metabolic processes involved in plant defense mechanisms of plants has suggested interpreting the effects of heat treatment through new metabolic pathways triggered by the natural antiviral response produced by the infected plant, with reference to Virus-Induced Gene Silencing (VIGS) [11]. Further investigations in infected plants found a correlation between VIGS and the temperature regimes and suggested a possible relationship between gene silencing and thermal increase [12,13]. The authors identified hyperactivity of the temperature-dependent gene silencing system as a mechanism of antiviral protection in the plant. In contrast, increased heat stress induces an increase in the host’s defense system capacity by creating a barrier to infection.

The present results support the potential of HWT as a promising sanitation strategy for dormant *Prunus* propagation material under the conditions described and indicate the possibility of integrating such treatments into routine nursery practices without incurring excessive additional costs. Further investigations are required to establish robust treatment protocols for routine commercial implementation, in particular larger-scale studies incorporating biological confirmation of pathogen viability, economic evaluation and validation across multiple cultivars and production systems.

## 4. Materials and Methods

The experimental protocol of thermotherapy involving hot water immersion was developed based on experimentation of the type of tissue and production operations at our nursery. Few studies are referenced in the literature on HWT for other plant species and different conditions [5,7].

### 4.1. Plant Material

For the experiments, almond (*Prunus dulcis*) and nectarine (*Prunus persica var. nucipersica*) plant material was used. The trials were carried out in two phases: (i) viability in dormant almond buds to determine their tolerance to different thermotherapy conditions, and (ii) thermotherapy trials to assess effectiveness against pathogens in almond material infected with *Prunus necrotic ringspot virus* (*PNRSV*) and nectarine material infected with *Xanthomonas arboricola pv. pruni* (*Xap*).

### 4.2. Pathogen Detection by RT-PCR

Prior to thermotherapy in pathogen-infected material, the presence and initial load of *PNRSV* and *Xap* were verified using real-time PCR method using a QuantStudio™ 3 Real-Time PCR System (Applied Biosystems, Thermo Fisher Scientific, Waltham, MA, USA).

#### 4.2.1. PNRSV Detection in Almond

A representative subsample corresponding to 10% of all budwood (240 buds) was processed. Total RNA was extracted using positively charged nylon membranes (Roche Diagnostics GmbH, a member of Merck KGaA, Darmstadt, Germany) following an established protocol. RNA was stabilized in glycine solution, and concentration and purity were assessed using a NanoDrop™ Lite Plus Spectrophotometer (Thermo Fisher Scientific, Wilmington, DE, USA). The samples were stored at −20 °C until real-time PCR analysis, performed following the protocol of Marbot et al. [14].

#### 4.2.2. Xap Detection in Nectarine

DNA extraction was performed using the Extract-N-Amp™ Plant PCR Kit and Extract-N-Amp™ Plant Dilution Solution (Sigma-Aldrich, St. Louis, MO, USA). DNA concentration and purity were assessed using a NanoDrop™ Lite Plus Spectropho-tometer (Thermo Fisher Scientific, Wilmington, DE, USA).

The detection of *Xap* was performed following both real-time PCR protocols described by Palacio-Bielsa [15] and Garita Cambronero [16].

### 4.3. Thermotherapy Treatments

In all cases, the almond buds were treated before grafting by the hot water immersion method using the Thermo RG R7Lab S2 equipment (RG Grapevine Nursery Machinery, Valencia, Spain) with capacity of 0.9 m^3^.

#### 4.3.1. Viability Assays in Almond

The first viability trial was conducted using four almond varieties: Avijor’, ‘Pentacebas’, ‘Vialfas’, and ‘Isabelona’. Five heat treatments were applied, using 96 dormant buds per treatment. The treatments evaluated were; 40 °C/30 min; 45 °C/30 min; 45 °C/40 min; 50 °C/40 min; control (untreated).

After treatment, all buds were grafted onto Rootpac^®^-20 rootstocks.

To further refine the conditions, a second viability test was conducted, this time using only ‘Avijor’ and ‘Pentacebas’ almond varieties. Nine treatments were applied, with 120 dormant buds per treatment: 45 °C/40 min; 45 °C/50 min; 45 °C/60 min; 48 °C/20 min; 48 °C/30 min; 48 °C/40 min; 50 °C/20 min; control (untreated).

Buds were grafted immediately after thermotherapy onto Rootpac^®^-20 rootstocks.

#### 4.3.2. Thermotherapy Treatments in Pathogen-Infected Material

Thermotherapy conditions were selected based on the results obtained in the viability trials.

In *Xap*-infected nectarines, three treatments were applied to batches of 48 dormant buds per treatment: 45 °C/20 min; 45 °C/40 min; control (untreated).

In the test *PNRSV*- infected almonds, four treatments were applied to batches of 240 dormant buds per treatment: 45 °C/50 min; 50 °C/20 min; 50 °C/30 min; control (untreated).

In both cases after thermotherapy, buds were micrografted onto the appropriate rootstocks; for nectarine buds were grafted into GF-677 and for almond buds were grafted into Rootpac^®^ R.

Twenty days after grafting, the rootstock apex was removed to promote bud break. Six weeks after grafting, the percentage of viable shoots was recorded to assess tolerance to heat stress.

Once bud break was complete, samples from each treatment were re-analyzed using RT-PCR to determine whether the thermotherapy reduced or eliminated the pathogen.

### 4.4. Statistical Analysis

Statistical analyses of the parameters analyzed in the treatments of *Prunus necrotic ringspot virus* and *Xanthomonas arboricola pv. pruni* infected *Prunus* species were performed using Jamovi software (Version 2.6.44; The jamovi Project, Sydney, Australia). Overall differences among treatments were assessed using Pearson’s chi-square test. When a significant overall effect was detected, comparisons between each thermotherapy treatment and the untreated control were performed using Fisher’s exact test with Holm adjustment for multiple comparisons. Additionally, 95% confidence intervals were calculated for each treatment using the Wilson method.

The statistical analyses described above were applied exclusively to plant viability and sprouting data. Pathogen detection results were obtained from pooled samples at the treatment level and were therefore interpreted descriptively.

## Figures and Tables

**Table 1:** RT-PCR results for *Xap* analysis obtained after treatments.

Treatment	Date of Analysis (Days After HWT)	RT-PCR Result for *Xanthomonas arboricola* *pv. pruni*
Untreated	16 July 2025 (97)	Positive (Ct value 33)
45 °C 20 min	16 July 2025 (97)	Weakly positive (Ct value 36)
45 °C 40 min	16 July 2025 (97)	Negative (no Ct value)

**Table 2:** RT-PCR results for *PNRSV* analysis obtained after treatments.

Treatment	Date of Analysis (Days After HWT)	RT-PCR Result for *Prunus necrotic ringspot virus*
Untreated	27 June 2025 (78)	Positive (Ct value 28)
45 °C 50 min	27 June 2025 (78)	Weakly positive (Ct value 31)
50 °C 20 min	27 June 2025 (78)	Positive (Ct value 27)
50 °C 30 min	27 June 2025 (78)	Non-sprouting plants

## Data Availability

The original contributions presented in this study are included in the article. Further inquiries can be directed to the corresponding author.

## Abbreviations

The following abbreviations are used in this manuscript:

*Xap*	*Xanthomonas arborícola pv. pruni*
*PNRSV*	*Prunus necrotic ringspot virus*
*Xf*	*Xylella fastidiosa*
DNA	Deoxyribonucleic acid
RNA	Ribonucleic acid
RT-PCR	Real-time–polymerase chain reaction
HWT	Hot water treatment
